# Approaching Alzheimer’s disease from a network level

**DOI:** 10.18632/oncotarget.14617

**Published:** 2017-01-13

**Authors:** Anna K. Gillespie, Emily A. Jones, Yadong Huang

**Affiliations:** Department of Neurology, Gladstone Institute of Neurological Disease, University of California, San Francisco, CA, USA

**Keywords:** Alzheimer’s disease, apoE, hippocampal network, sharp wave ripple, slow gamma, Neuroscience

Despite decades of research, the root “cause” of Alzheimer’s disease (AD) remains elusive. Historically, most AD research has focused on genes implicated in early onset AD, such as amyloid precursor protein and the presenilins. However, these cases comprise less than 1% of all AD cases. The vast majority of AD cases are late onset and of unknown etiology, and in these cases, another genetic risk factor dominates: apolipoprotein E4 (apoE4). ApoE4 carriers, especially women, have an increased risk of developing AD and a lower average age of onset compared to carriers of the more common isoform, apoE3 [1]. Although the impact of apoE4 is clear, the pathological mechanisms underlying the increased AD risk are not well understood.

On a molecular level, a single amino acid substitution differentiates apoE4 from apoE3, altering its structure, interfering with its normal function, and rendering it vulnerable to proteolytic cleavage into toxic fragments. To study the detrimental effects of apoE4 in an animal model, our lab and others use knock-in (KI) mice that express human apoE4 at the endogenous mouse apoE locus. Using these mice, we have shown that inhibitory interneuron populations in the dentate gyrus of the hippocampus of aged, female apoE4-KI mice are particularly vulnerable to apoE4 expression. In addition to this cellular phenotype, aged female apoE4-KI mice also show impairment on a hippocampus-dependent learning and memory task, which is correlated with the extent of interneuron loss [2]. Further studies provided additional evidence for a causal relationship between interneuron loss and behavioral impairment[3, 4], however, the functional link between the two was unclear.

To address this question, we looked to the network activity in which these interneurons likely participate. While many studies have linked patterns of hippocampal activity to normal memory processes, the characterization of these hippocampal network-level patterns in disease models has been relatively rare. We were particularly interested in sharp-wave ripples (SWRs), brief high frequency oscillations in the hippocampal local field potential signal. These events are notable because they coincide with hippocampal replay, a phenomenon in which ensembles of hippocampal neurons are reactivated in a manner that recapitulates prior experience. As such, hippocampal replay during SWRs is thought to be critical for memory consolidation and potentially retrieval [5]. We hypothesized that apoE4-induced interneuron malfunction might alter SWR activity, disrupting mnemonic processing and thus causing memory impairment.

Indeed, we found that compared to apoE3-KI mice, apoE4-KI mice have fewer SWRs. Beyond that, the remaining apoE4-KI SWRs showed reduced power in a coincident lower frequency oscillation called slow gamma. As slow gamma during SWRs is thought to be a critical coordinator of accurate replay [6], both the reduction in replay events and the potentially degraded quality of replay due to slow gamma attenuation were promising candidate mechanisms for the apoE4-induced behavioral deficit. To further link the functional phenotype with our prior findings of interneuron loss in the dentate gyrus, we used a line of mice with the apoE4 gene removed specifically in inhibitory interneurons (apoE4-KI/Dlx- Cre mice). This genetic manipulation prevents both interneuron loss and learning and memory impairment [7]. Interestingly, the apoE4-induced reduction in SWR events was maintained in these mice, while the attenuation of slow gamma activity during SWRs was prevented. This suggests that the slow gamma activity during SWRs is dependent on intact interneuron activity and is critical for learning and memory processes. Studies of young apoE4-KI mice - prior to extensive interneuron loss and behavioral deficits - also showed a reduction of SWR events, further corroborating that the reduction of SWR events alone is insufficient to disrupt behavior. In contrast, the slow gamma during SWRs showed an age-dependent, progressive decline, aligned with the development of behavioral impairment and loss of interneurons [8].

Together, these results provide important insights to both normal hippocampal mnemonic processing as well as the pathological mechanisms of apoE4 in AD pathogenesis. We suggest that attenuated slow gamma activity during SWRs, rather than the reduction in SWR events, is likely to drive learning and memory impairment in apoE4-KI mice; the *quality* of SWRs may be more critical than their *quantity* in maintaining mnemonic function. Further, we conclude that interneuron populations, particularly those in the dentate gyrus, may be integral to maintaining slow gamma oscillations throughout the hippocampal circuit.

These findings have important implications for designing therapeutic strategies for combating AD and evaluating current therapeutic candidates. One appealing solution under development uses small molecules to correct the protein structure of apoE4 to a more apoE3- like conformation, reducing the production of toxic fragments from the proteolysis of apoE4. This may protect interneurons from apoE4’s detrimental effects and promote their function, since these inhibitory populations are particularly vulnerable to apoE4 pathologies. Alternatively, drugs that strengthen GABAergic signaling, especially ones specifically targeting the dentate gyrus, may be able to protect against or compensate for reduced interneuron function. Both the apoE4 structure corrector and the GABAergic signaling enhancer, as well as any other therapeutic candidates for AD, could benefit from using network activity, such as SWR-associated slow gamma, as a biomarker. This network-level phenotype may serve to monitor progression of AD symptoms over aging in mouse models or reflect successful modulation of hippocampal function by therapeutic compounds.
